# Transoral ultrasonic total laryngectomy (TOUSS-TL): description of a new endoscopic approach and report of two cases

**DOI:** 10.1007/s00405-015-3784-5

**Published:** 2015-10-01

**Authors:** Mario M. Fernández-Fernández, Lourdes Montes-Jovellar González, Carlos Ramírez Calvo, Pablo Parente Arias, Francisco Clascá Cabré, Primitivo Ortega del Álamo

**Affiliations:** 1 Department of Otorhinolaringology, Henares University Hospital, c/Marqués de Lozoya no 3-escC-9oB, 28007 Coslada, Madrid Spain; 2 Department of Head and Neck Surgery, MD Anderson Cancer Center Madrid, c/Arturo Soria 270, 28033 Madrid, Spain; 3 Department of Otorhinolaringology, VITHAS Nuestra Señora de América Hospital, c/Arturo Soria 103, 28033 Madrid, Spain; 4 Department of Otorhinolaringology, Complexo Hospitalario Universitario de A Coruña, c/As Xubias 84, 15006 A Coruña, Spain; 5 Anatomy and Neuroscience Department, Autonoma University Medical School, Madrid, Spain; 6 Department of Otorhinolaringology, Mostoles University, c/Río Júcar s/n, 28935 Móstoles, Madrid Spain; 7 Hospital Universitario del Henares, Servicio de Otorrinolaringología, Avda Marie Curie s/n, 28822 Coslada, Madrid Spain

**Keywords:** TOUSS, TORS, Transoral robotic surgery, Total laryngectomy, Transoral surgery, Minimally invasive surgery

## Abstract

The minimally invasive total laryngectomy avoids a wide surgical field and so it has the potential benefit of reducing the local morbidity, especially on radiated patients. This approach has been previously described on a robotic basis, the transoral robotic total laryngectomy (TORS-TL). We have designed a minimally invasive approach for total laryngectomy (TL) using the transoral ultrasonic surgery technique (TOUSS). TOUSS is a transoral, endoscopic, non-robotic approach for laryngeal and pharyngeal tumors, based on the ultrasonic scalpel as a resection tool. Two patients with a laryngeal squamous cell carcinoma with indication for total laryngectomy were surgically treated: one primary TL for a subglottic carcinoma and one salvage TL with partial pharyngectomy for a local relapse after chemoradiotherapy of a glottic carcinoma. The tumors were completely removed with free surgical margin in both patients. The functional recovery was satisfactory in terms of swallowing and speech (a tracheoesophageal puncture and voice prosthesis placement were done in the same procedure). No intraoperative complications were observed. The patient with previous chemoradiotherapy had a pharyngocutaneous fistula which closed spontaneously without additional surgery. We have demonstrated that transoral endoscopic approach to the larynx and pharynx is feasible without a robotic platform. TOUSS-TL can easily spread the transoral endoscopic philosophy as well as the benefits of a minimally invasive way to remove the entire larynx. Further research will show the advantages in terms of complications and functional outcomes.

## Introduction

The recent interest in transoral approaches states a concern about the sequelae and functional impact of chemoradiation, open surgical techniques, and salvage surgery. Transoral robotic surgery (TORS) has shown its good oncological and functional results over the past years [1–4]. In fact, the potential of the transoral robotic approach for pharyngeal and laryngeal cancer treatment has led to an expansion of its indications. The combination of supraglottic and hypopharyngeal transoral approach allowed the description of the transoral robotic total laryngectomy (TORS-TL) [5, 6]. However, most of the head and neck surgical teams cannot start with the transoral endoscopic approach as the robotic platform remains unreachable for many institutions. Transoral ultrasonic surgery (TOUSS) has been described in 2014 as a transoral endoscopic approach initially for oropharyngeal, hypopharyngeal and supraglottic tumors [7]. The main advantage of TOUSS is the achievement of the same output as TORS without the costs of a robotic platform. We also hypothesized that the avoidance of neck scars, musculocutaneous flaps, and the reduction of pharyngotomy size, should offer a significant benefit for the patient in terms of morbidity. In the present report, we describe the surgical technique for a transoral endoscopic total laryngectomy by using a TOUSS setup, and the preliminary clinical experience with the technique.

## Materials and methods

The transoral endoscopic ultrasonic total laryngectomy (TOUSS-TL) surgical technique has been established on cadaver basis in the anatomy dissection lab. The open technique surgical steps were adapted to an endoscopic approach. Once the technique was well established and fully satisfactory on a cadaver, the indications for it were aimed at avoiding neck incisions. The protocol to treat human subjects with TOUSS-TL was approved by our institutional review board. The inclusion criteria were (1) at least 18 years old, (2) laryngeal cancer with indication for total laryngectomy, (3) laryngeal cancer without indication for neck dissection, (4) and consent for transoral ultrasonic total laryngectomy. Exclusion criteria were (1) pregnancy, (2) unable to understand the surgical procedure, (3) no invasion through the thyroid cartilage or soft tissues of the neck (T4a) or major extralaryngeal invasion (T4b). Further studies will establish the advantages of transoral total laryngectomy simultaneous or in combination to neck dissection. All patients were counseled about open alternatives and non-surgical strategies (when indicated) and informed consent was obtained from all of them for a minimally invasive approach to their laryngeal cancer.

### Materials

The OR setup and surgical instruments were defined for TOUSS [7]. The Feyh-Kastembauer retractor is used for the transoral exposition of the larynx. The Olympus ENDOEYE™ 10 mm 3D and 5 mm 2D videoendoscopes were used in combination with the Martin’s arm scope holder. The deflecting tip properties of ENDOEYE™ videoendoscopes allow a fine tuning of the endoscopic surgical view using the joystick adjustments at the camera head. The 35 cm Thunderbeat™, an integrated ultrasonic and bipolar cutting-coagulating device, is used as resection tool. Besides the ultrasonic scissors, Thunderbeat integrates a bipolar vascular sealing system approved for safely sealing vessels up to 7 mm [8]. A long aspiration cannula is used by the assistant to keep a clean endoscopic view, removing the aerosol released during the activation of the ultrasonic scalpel. For more delicate areas like mucosa over the arytenoid cartilages or the lingual aspect of the epiglottis, a long monopolar needle electrode for endoscopic laryngeal surgery was used. Finally, a complete set of laparoscopic instruments is required to help in transoral resection of the larynx.

### Surgical technique

**Fig. 1:**
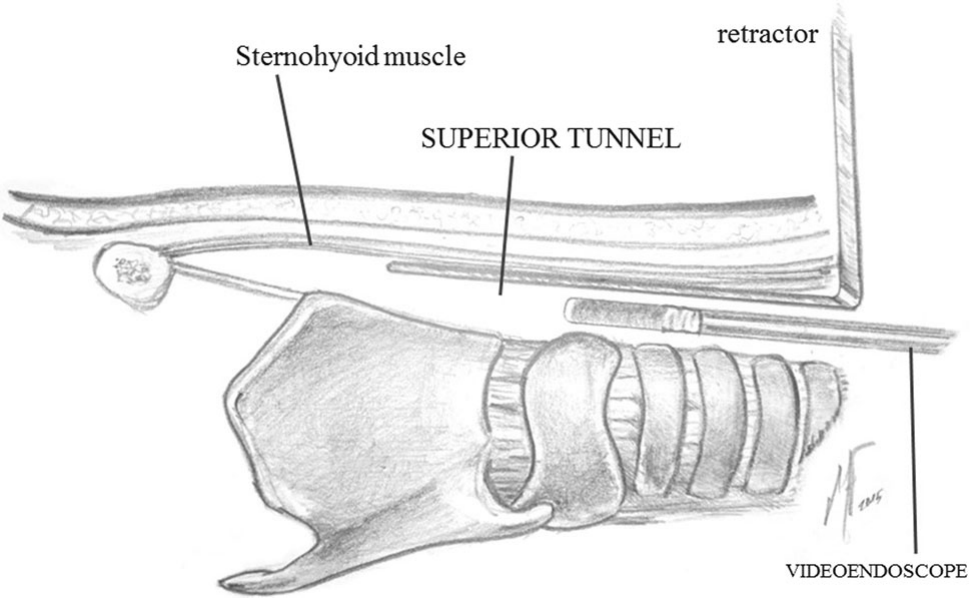
Lateral view of the superior tunnel. The videoendoscope is introduced through the cervical incision, in the space under the sternohyoid muscle

**Fig. 2:**
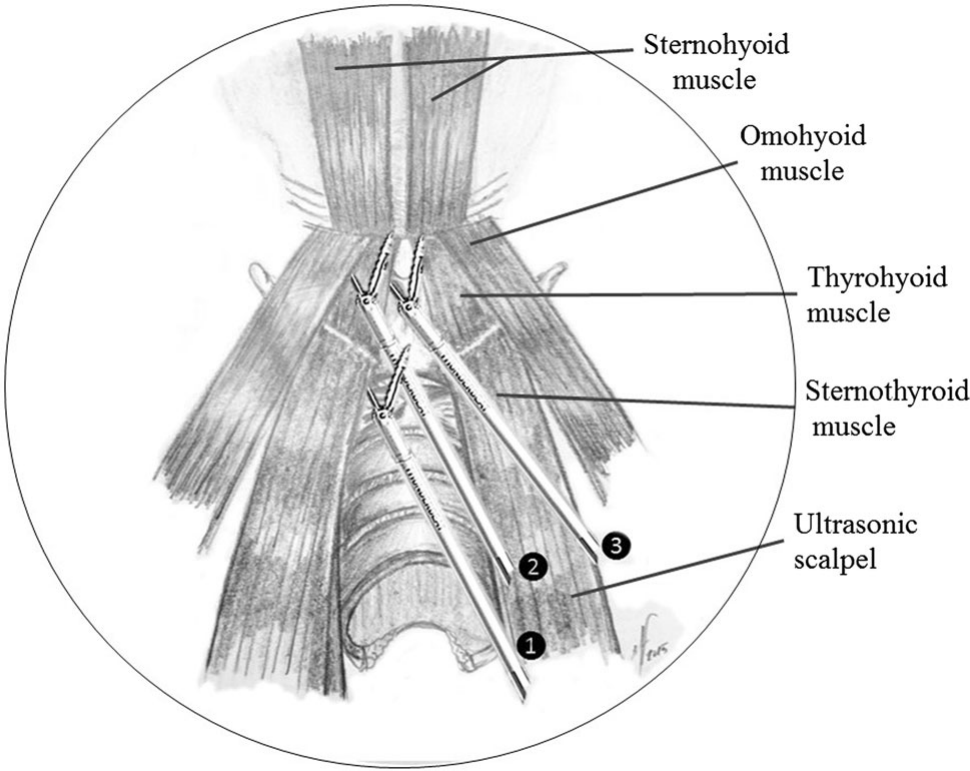
Endoscopic view of the superior tunnel. The endoscopic transection of the sternothyroid (*1*), omohyoid (*2*) and thyrohyoid (*3*) muscles is done close to their superior insertion by using the ultrasonic scalpel
Step 5: **Inferior tunnel**.
  The trachea is now opened and the tracheal tube is placed through the tracheostoma. Three stitches are given between the trachea and the skin. Then, the posterior wall of the trachea is incised at the level of the superior ring, obtaining a beveled-cut tracheostoma. This will contribute to obtaining a wider tracheostoma as well as a better positioning of the voice prosthesis. The inferior tunnel is started dissecting the trachea from the esophagus while the trachea is pulled upwards using a retractor. Care must be taken again to avoid reaching the tumoral lesion. The posterior cricoarytenoid muscles are exposed and the posterior aspect of the arytenoid cartilages is reached. Blunt dissection is desirable as well as careful coagulation. Some blunt dissection of the inferior aspect of the pyriform sinus can be done as well, and blind maneuvers should be avoided. The constrictor muscle is sectioned laterally at the level of the posterior border of the thyroid cartilage up to the superior cornu. The superior cornu is also released as much as possible. A piece of gauze can be left in the retrocricoid area in order to facilitate the transoral endoscopic opening of the mucosa of the inferior tunnel (Figs. 3, 4).

Step 1: **Patient positioning**.
  The patient is placed in supine position without elevation of shoulders. The avoidance of neck extension will facilitate the generation of much wider neck surgical field.
Step 2: **Cervical incision and thyroid gland exposure**.
  A 3–4 cm central horizontal incision is done 2 cm above the sternal notch, taking into account the final position of the tracheostoma. The superficial cervical fascia is incised and anterior jugular veins are transected with Thunderbeat™. The strap muscles are also transected at this level and sternohyoid muscles are sutured to the musculocutaneous flap. Now, the isthmus of the thyroid gland is exposed.
Step 3: **Thyroid isthmus section and tracheal exposure**.
  The isthmus of the thyroid gland is transected using the ultrasonic cutting mode of Thunderbeat™. Next, the thyroid lobes are separated laterally from the trachea and the larynx using also the ultrasonic scalpel in order to avoid small bleeding, especially at the level of the cricoid artery on the medial aspect of the thyroid lobe.
Step 4: **Superior tunnel**.
  The dissection continues in a minimally invasive endoscopic fashion through the cervical incision using the 5 mm videoendoscope. The superior tunnel is the space created under the sternohyoid muscles up to the level of the hyoid bone. The first assistant keeps opened the superior tunnel with a Langenbeck retractor while a second assistant holds the videoendoscope through the incision and exposes the surgical field. Once the superior tunnel is finished, the superior aspect of the sternothyroid muscle is transected on each side of the larynx using the Thunderbeat™. This will expose completely the superior thyroid lobe, so it can be now completely released laterally from the larynx. The tunnel is progressed superiorly sectioning the omohyoid muscle and the inferior insertion of the thyrohyoid muscle (Figs 1, 2).

**Fig. 3:**
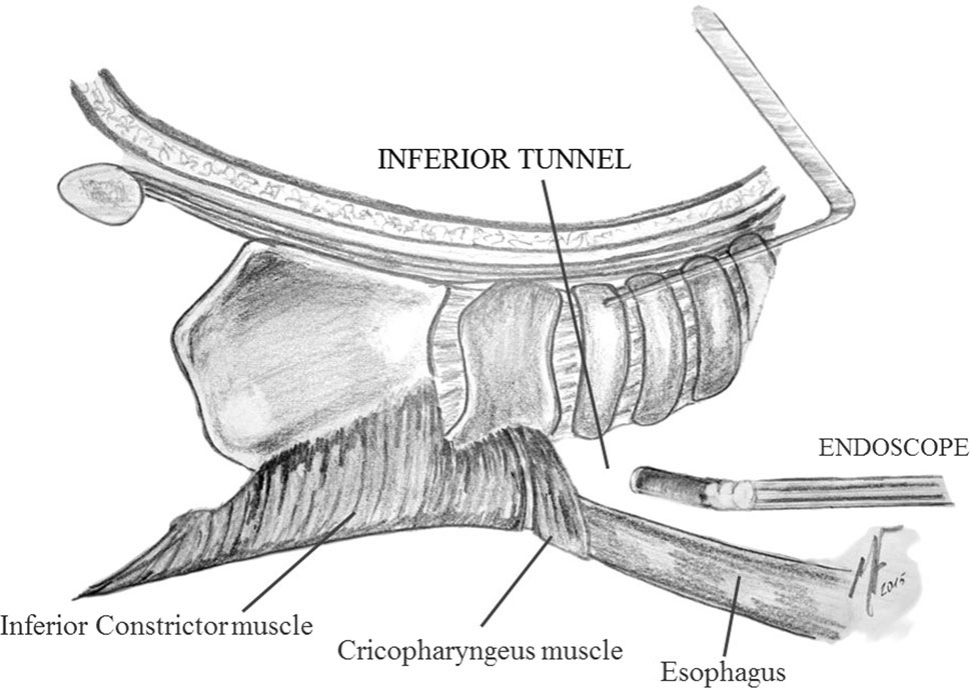
Lateral view of the inferior tunnel. The larynx is dissected up to the level of the arytenoid cartilages

**Fig. 4:**
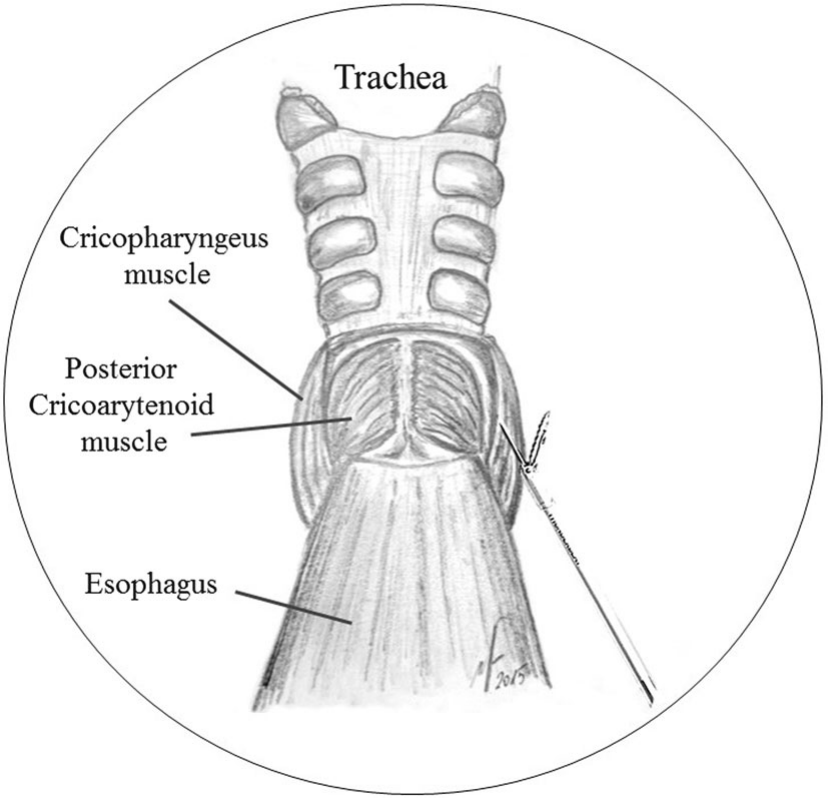
Endoscopic view of the inferior tunnel, and exposure of the posterior cricoarytenoid muscles
Step 6: **Transoral approach**.
  The larynx is exposed transorally with the FK-retractor. The videoendoscope is attached to the scope holder. Retraction of the epiglottis can be necessary for exposing the retrocricoid area when the surgical margin runs in the postcricoid area. The limits of the mucosa resection are marked with the long needle electrode. The valleculae can be incised directly using the Thunderbeat™. However, if preservation of the mucosa of lingual aspect of the epiglottis is required, the incision should begin with the needle electrode in order to avoid excessive damage on the epiglottic cartilage. If a piece of gauze was left in the retrocricoidspace, the incision should be done towards it. The monopolar needle electrode can better preserve the mucosa of the arytenoid cartilage and postcricoid area compared with Thunderbeat™. The posterior mucosal dissection progresses laterally towards the anterior incision, and in deeply towards the inferior tunnel (Fig. 5).

**Fig. 5:**
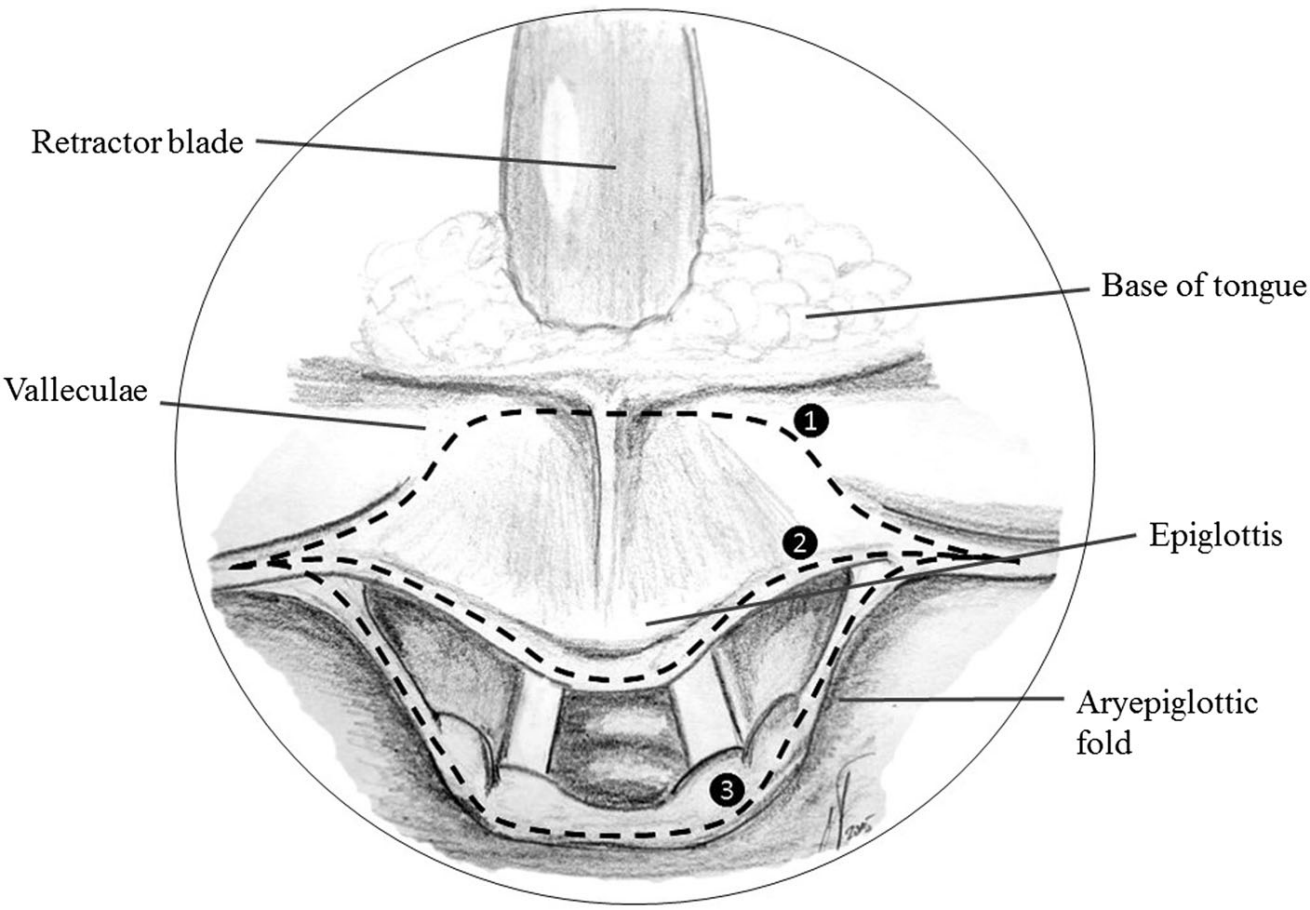
Endoscopic transoral approach of the larynx. Section of the mucosa of the valleculae (*1*) is done with the ultrasonic scalpel. If mucosa of the lingual aspect of the epiglottis can be preserved, the section (*2*) should be incised with the monopolar electrode, as well as the posterior section of the mucosa (*3*)
Step 7: **Transoral anterior approach**.
  The FK-blade is placed behind the base of the tongue facilitating the transoral exposure to the preepiglottic space. Progression towards the inferior border of the hyoid bone is easily done with the Thunderbeat™. At this point, one assistant should compress downwards the larynx to facilitate the exposure. The superior tunnel is easily entered and dissection is conducted laterally towards the superior cornu of the thyroid cartilage. A careful progression with Thunderbeat™ will ensure an effective coagulation of the superior laryngeal pedicle. A lateral enlargement of the mucosal incision can be necessary if the release of the superior cornu couldn’t be completed through the inferior tunnel (Fig. 6).

**Fig. 6:**
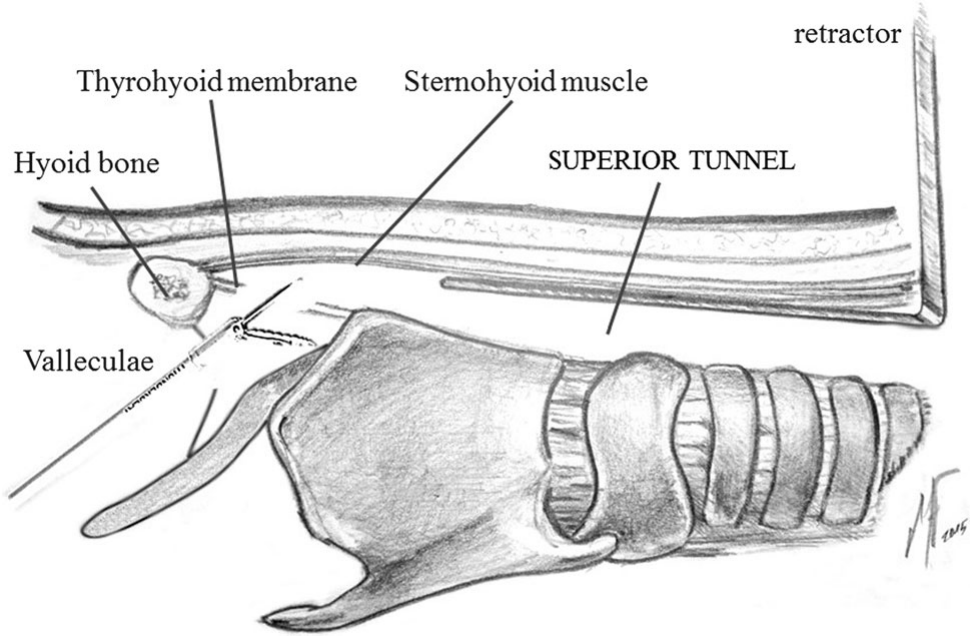
Lateral view of the transoral infrahyoid resection. The section of the preepiglottic space runs under the hyoid bone and enters the superior tunnel
Step 8: **Transoral resection of the larynx**.
  Finally, traction of both superior cornu is made with two forceps and the larynx is completely removed transorally.
Step 9: **Pharyngeal reconstruction**.
  A continuous suture line is given from the lateral side of the mucosal defect using 3/0 absorbable monofilament suture. The pharyngeal closure runs horizontally to completely close the pharyngeal mucosa. If the postcricoid mucosa cannot reach the base of the tongue due to the extension of the mucosal resection, the pharyngeal closure should be done against the strap muscles. Now, a nasogastric feeding tube can be placed before removing the FK-laryngopharyngoscope (Fig. 7).

**Fig. 7:**
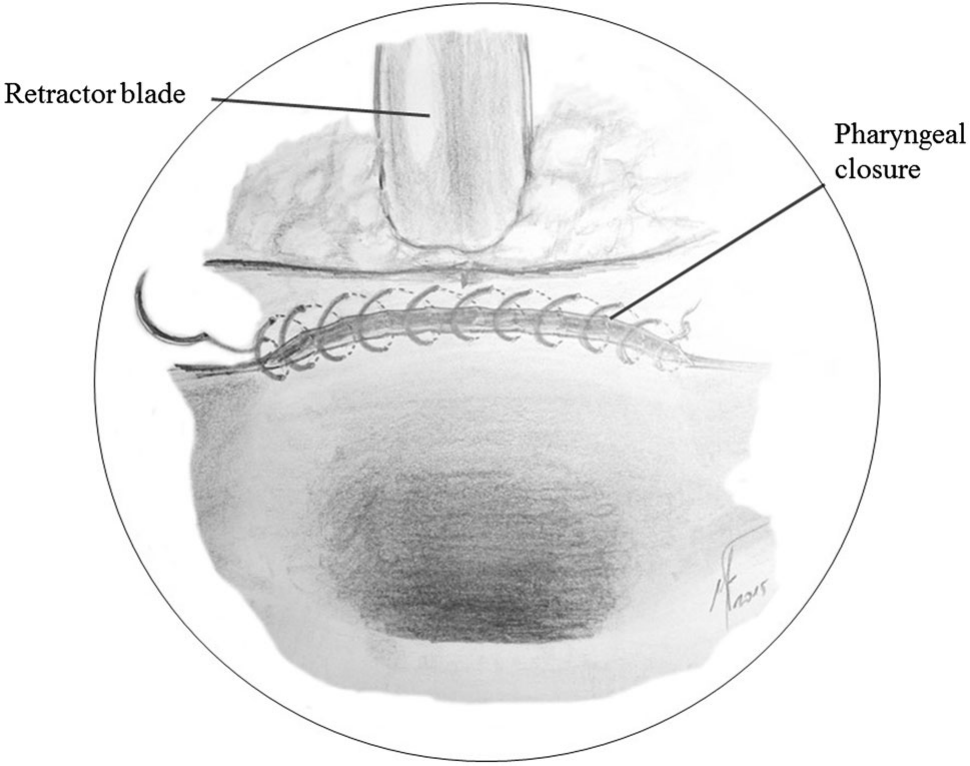
Pharyngeal closure. The pharynx is closed using a continuous suture
Step 10: **Cervical incision closure (Tracheostoma)**.
  Two small drainages are placed laterally to the esophagus. Patients with wide resection of retrocricoid or pharyngeal mucosa or minimal tracheal resection might not have enough neck space, and no drainages are required. The musculocutaneous flap is sutured to the anterior pharyngoesophageal wall to improve the contact between layers, and tracheostoma is performed. Now, a tracheoesophageal fistula can be performed as a secondary puncture and a voice prosthesis is fitted into the fistula. Neck compression bandage should be maintained during 5 days on non-radiated patients, and 7 days on radiated patients.

## Results

Two patients were treated for their laryngeal carcinoma with TOUSS-TL between February and March 2015. Patient #1 was a 61-year-old male patient scheduled for a salvage total laryngectomy after a local relapse of a T3N1M0 left supraglottic squamous cell carcinoma. The patient was treated with chemoradiotherapy. The recurrent tumor affected the left aryepiglottic fold and left arytenoid cartilage with vocal cord fixation. There was no evidence of thyroid cartilage infiltration or suspicious neck nodes on CT scan. Counseling about transoral ultrasonic total laryngectomy and open technique was given to the patient and he consented a TOUSS-TL technique. After general anesthesia, OR set up for TOUSS was done in 5 min. The total surgical time was 210 min. An extralaryngeal superficial extension to the right piriform sinus was identified during the surgery so a wide resection of right piriform sinus and right pharyngeal wall was mandatory to adequate the surgical margin. The retrocricoid tumoral extension required also a wide mucosal resection so direct mucosal closure was not possible. The pharyngeal closure was done against the anterior musculocutaneous wall and the sternohyoid strap muscles. Finally, a tracheoesophageal puncture using a secondary puncture set was done and a voice prosthesis (Provox^®^) was fitted in the fistula. There was no worthy bleeding during surgery and no blood transfusions were necessary at any time. Postoperative pain was controlled with corticosteroids and one non-steroidal anti-inflammatory drug. Surgical margins were negative for malignant cells. In the day after surgery, a tongue inflammation was evident due to the prolonged used of Kastembauer retractor. A progressive reduction in tongue inflammation was observed after 1 week, and it came back to normal 3 weeks after the onset. A pharyngocutaneous fistula was evident on day 8 after releasing the neck bandage. Compressive neck bandage was maintained and the fistula was completely closed was in 7 weeks. The patient is satisfactorily swallowing and a wide neopharynx is evident in the barium esophagogram study. The patient is using normally his prosthetic voice. The final result is an optimal neck status without any discomfort, despite the radiated status of the tissues.

Patient #2 was a 74-year-old male patient diagnosed with a T2 subglottic squamous cell carcinoma. Clinical exploration and CT scan revealed no suspicious neck nodes. The patient was counseled about the surgical and nonsurgical treatment options. Finally the patient consent to minimally invasive total laryngectomy and transoral endoscopic total laryngectomy (TOUSS-TL) was scheduled as primary treatment. The OR set up was done in 5 min, and the total laryngectomy including the pharyngeal closure was completed in 180 min. No 3D equipment was used in this particular case. After pharyngeal closure, a tracheoesophageal puncture was done using a rigid esophagoscope and a secondary puncture set. A Blom-Singer voice prosthesis was fitted in the tracheoesophageal fistula. No intraoperative or postoperative blood transfusions were necessary. In fact, during the surgical procedure there was no worthwhile bleeding. Surgical margins were free of tumor and final pathological classification was T2N0M0-II. Oral intake was started on day 6 and fully oral diet began on day 13 and so the patient could be discharged from the hospital. The nasogastric feeding tube was maintained until a fully oral diet was possible. Postoperative pain was controlled with one non-steroidal anti-inflammatory drug. The patient is using tracheoesophageal voice regularly.

**Fig. 8:**
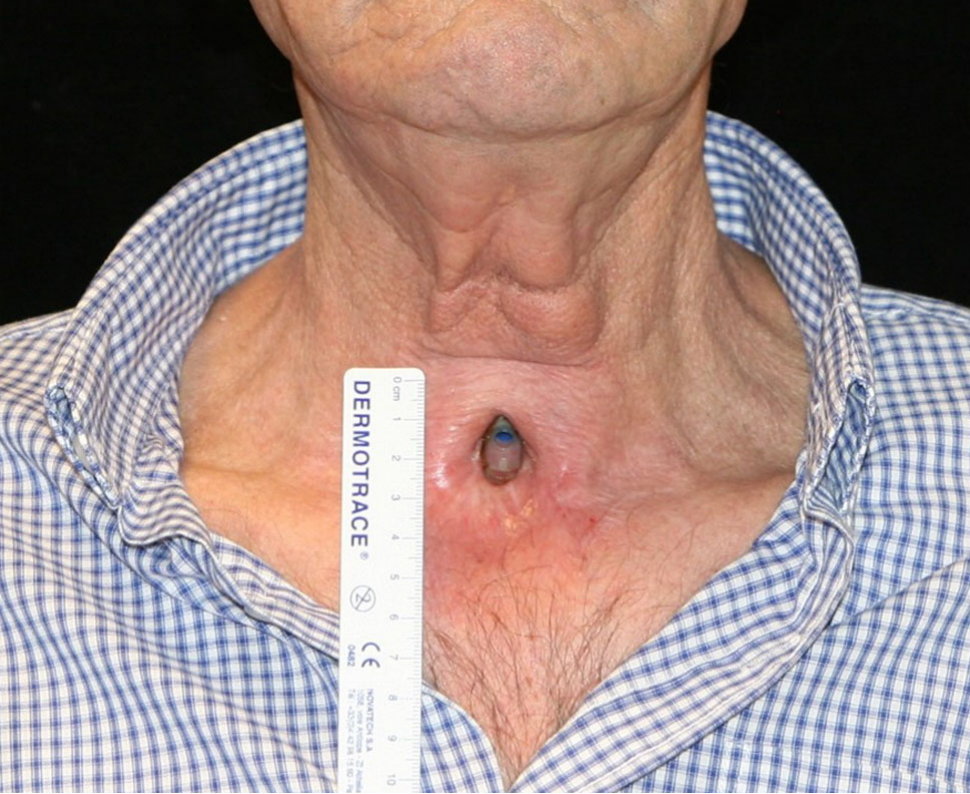
Wide neopharynx and optimal swallowing in esophagogram

**Fig. 9:**
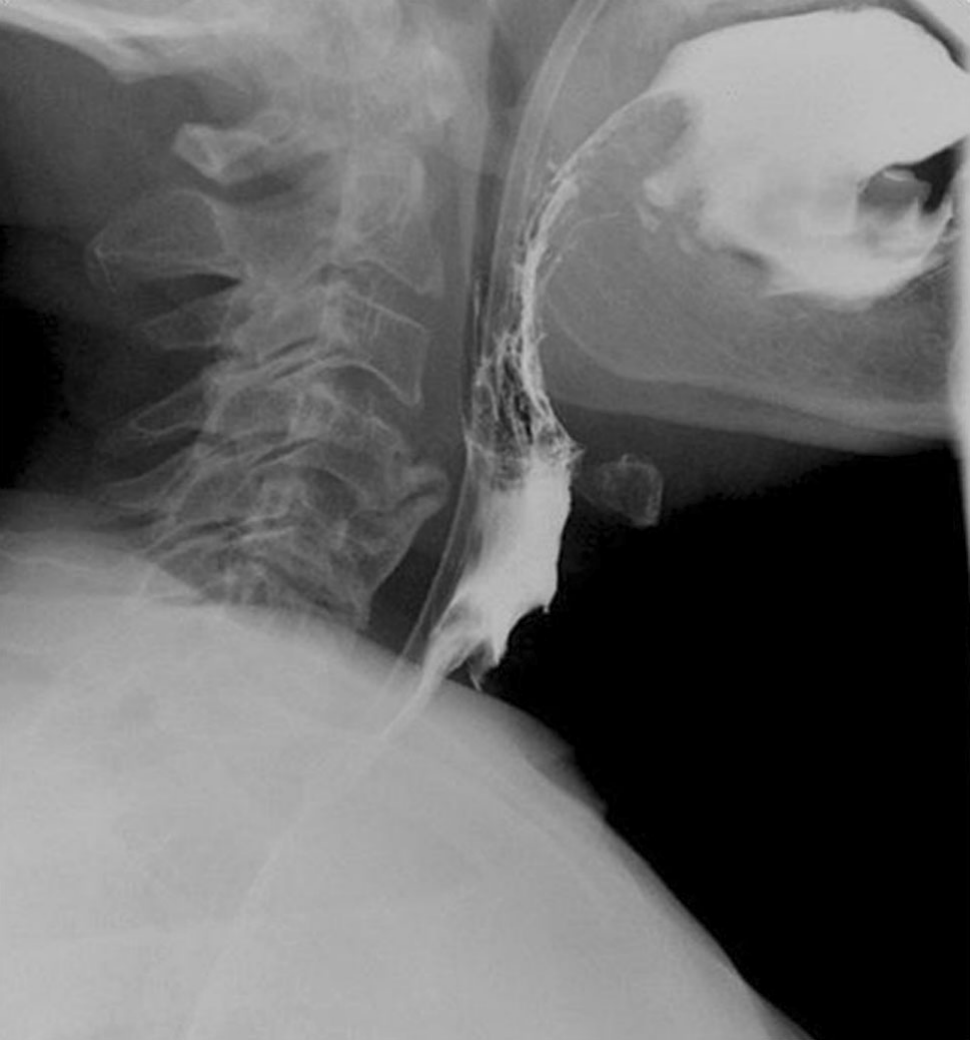
No neck scars and optimal conditions of neck skin without local morbidity derived from flap elevation

The final result, a wide neopharynx on the barium pharyngoesophagogram (Fig. 8), and optimal neck skin conditions (Fig. 9) are shown in the figures.

## Discussion

The improvements in imaging equipment technology, as well as better cutting-coagulating instruments, have led to a new era in minimally invasive surgery. Since robotic surgery opened the door to transoral endoscopic approaches for laryngeal and pharyngeal tumors, the complexity of the procedures has also increased [1–4]. Even advanced tumors and large defects have not been a problem from a reconstructive point of view after a transoral robotic resection [9]. Recently, TOUSS has been described by our group as a transoral endoscopic surgical technique to treat supraglottic or pharyngeal cancer avoiding the need for a robotic platform [7].

Despite the decrease of primary indications of total laryngectomy, it still has a major role in the treatment of advanced laryngeal cancer. The increased number of organ preservation protocols has led to a raising number or salvage total laryngectomies. Salvage open total laryngectomy usually carries a higher incidence of early and late complications [10]. We can hypothesize some advantages of a minimally transoral technique for total laryngectomy compared to an open conventional technique. The avoidance of neck incisions and flap elevation, as well as the reduction in pharyngotomy size, has the potential benefit of lowering the local morbidity and complications on radiated patients. So, in 2013, Lawson et al. described the surgical steps for TORS-TL [5]. The aim of this minimally invasive, robotic approach for total laryngectomy was to reduce the size of the pharyngotomy and to limit the exposure of deep cervical structures [11]. Following this paper, only a few studies have been published with further experience by the technique [6, 12]. In fact, the higher costs of robotic total laryngectomy were highlighted by Dombree et al. [13]. The robotic platform increases the costs of the procedure almost twice compared to open total laryngectomy. Additionally, it is also important to take into account the high costs of acquisition of the robotic equipment. We have defined the surgical steps for a non-robotic minimally invasive total laryngectomy using only conventional laparoscopic equipment. The procedure basically consists of a combination of a minimally invasive endoscopic cervical approach through the tracheostomy skin incision, and a transoral endoscopic approach. The section of the infrahyoid and constrictor muscles through the inferior cervical incision allows an easy transoral removal of the larynx after the dissection of the supraglottic tissues. We can hypothesize a faster procedure with 3D vision as it allows a better endoscopic spatial positioning. However, the advantages of 3D imaging for transoral approach compared to 2D imaging are still under evaluation. In our study, case #2 was done with 2D equipment in less operating time than case #1 with 3D imaging. The better outcome of case #2 in terms of complications is probably related to the already radiated neck tissues of case #1, and the extralaryngeal mucosal extension that determined a wide pharyngeal resection and a longer procedure. The complete release of the laryngeal muscular attachments (infrahyoid and constrictor muscles) from the inferior cervical incision has facilitated the following transoral resection. This aspect was critical in reducing surgical time in the second patient.

Robotic TL seems to fit only in selected cases. However, it is considered a promising technique for the future of total laryngectomy [11]. In our belief, radiated patients benefit the most as it minimizes incisions and neck exposure. Neck dissection in salvage surgery is based on CT scan findings, and patients staged as *N*_0_ radiologically are unlikely to harbour occult nodal disease [14]. So, salvage total laryngectomy without neck dissection is the most relevant indication of this technique from this point of view. But when neck dissection is mandatory, the benefits of transoral total laryngectomy do not seem to be so remarkable as the neck incisions are already required.

In this study, we present two patients who underwent a successful TOUSS-TL. The tumors were removed with free surgical margins and a complete functional recovery was observed in both patients. Patient #1 had a pharyngeal extension of the tumor, so a wide resection of the right piriform sinus and lateral pharyngeal wall was required to remove the entire lesion. Due to such an extension of the lesion and the previous radiotherapy, the patient developed a pharyngocutaneous fistula and a delay for oral feeding was required. The incidence of pharyngocutaneous fistula increases dramatically on radiated patients and it is considered an independent prognostic factor [15]. However, the patient recovered only with conservative treatment and no additional neck surgery (free or pedicled flap) was necessary. Additionally, the horizontal fashion pharyngeal closure against the musculocutaneous flap due to the large mucosal defect allowed a wide pharyngeal lumen instead a narrow pharynx as the result of the conventional pharyngeal closure. Patient #2 had a T2 subglottic carcinoma and surgical treatment allowed the preservation of all pharyngeal mucosa.

As it was reported for TORS-TL, the horizontal pharyngeal defect after TOUSS-TL is shorter compared to open total laryngectomy [11]. Furthermore, these techniques avoid the T-shaped closure and trifurcation which is well known as a weak aspect of the suture, especially on radiated patients. If an extended pharyngectomy is needed, the pharynx can be closed using the strap muscles, achieving a wide pharyngeal lumen and avoiding a narrow neopharynx. The limited neck exposure could reduce the pharyngocutaneous fistula rate on radiated patients and systematic reinforcement of the suture with regional or free flaps.

Microscopic laser surgery has led the transoral surgical treatment for laryngeal tumors, even for oropharyngeal and hypopharyngeal lesions [16–18]. However, its coagulating properties are poor since only 0.5 mm vessels can be safely sealed, especially compared with ultrasonic scalpel [19]. So it does not seem to be the adequate instrument to get a bloodless endoscopic surgical field in such a wide resection as a total laryngectomy. The ultrasonic scalpel has not been described as a resection tool for TORS [20], probably due to the straight design and the lack of bendable properties. However, it is one of the critical aspects in TOUSS procedure. TOUSS has shown a great potential to become an alternative to TORS even for wide resections like a total laryngectomy.

## Conclusion

Transoral endoscopic ultrasonic minimally invasive total laryngectomy (TOUSS-TL) is feasible, and it can reduce the morbidity of an open total laryngectomy especially in salvage surgery. TOUSS-TL is a promising way to easily spread the minimally invasive approach to radical laryngeal surgery. The avoidance of expensive equipment takes TOUSS-TL within the reach of most head and neck surgical teams and institutions. So more patients can benefit from minimally invasive approaches to head and neck cancer. Further research will show the advantages in terms of complications and functional outcomes.
